# SAluMC: Thwarting Side-Channel Attacks via Random Number Injection in RISC-V

**DOI:** 10.3390/e27020202

**Published:** 2025-02-14

**Authors:** Shibo Dang, Yunlong Shao, Zhida Li, Adetokunbo Makanju, Thomas Aaron Gulliver

**Affiliations:** 1 College of Engineering and Computing Sciences, New York Institute of Technology, Vancouver, BC V5M 4X3, Canada; sdang@nyit.edu (S.D.); zli74@nyit.edu (Z.L.); amakanju@nyit.edu (A.M.); 2 Department of Electrical and Computer Engineering, University of Victoria, Victoria, BC V8W 2Y2, Canada

**Keywords:** RISC-V microarchitecture, side-channel attack, random number, attack countermeasure

## Abstract

As processor performance advances, the cache has become an essential component of computer architecture. Moreover, the rapid digital transformation of daily life has resulted in electronic devices storing greater amounts of sensitive information. Thus, device users are becoming more concerned about the security of their personal information, so improving processor performance is no longer the sole priority. Hardware vulnerabilities are generally more difficult to detect and address compared to software viruses and related threats. A common technique for exploiting hardware vulnerabilities is through side-channel attacks. They can bypass software security to extract personal information directly from hardware components like the cache or registers. This paper introduces a novel architecture for the arithmetic logic unit (ALU) and associated memory controller (MC) based on the RISC-V microarchitecture to mitigate side-channel attacks. The proposed approach employs hardware-generated random numbers and has minimal design costs, negligible impact on the original system structure, seamless integration, and easy modification of internal components. Results are presented that show it is effective against side-channel attacks.

## 1. Introduction

Electronic devices have become an integral part of daily life. As a consequence, an increasing number of people store confidential or sensitive information on devices like PCs and smartphones. Even devices that may seem to hold no personal data, such as the engine control unit (ECU) in automobiles, often contain sensitive information that can easily be overlooked. As a result, safeguarding the confidentiality of information is a crucial concern for both users and designers.

The increase in internet and electronic device usage has made cybersecurity a serious issue. As a consequence, software, network, and web security have attracted significant attention. This includes cache-based side-channel attacks, countermeasures, and subsequent counterattacks. In 2017, attacks related to many modern processors were discovered. In particular, Intel and ARM processors are vulnerable to an attack called Meltdown [1], and Intel, AMD, and ARM processors are vulnerable to an attack called Spectre [2]. Hardware security has long been a critical concern, especially as computing systems have become increasingly integrated into daily life and critical infrastructure. Hardware security threats can arise during various stages of the semiconductor life cycle. Side-channel attacks have emerged as a significant bottleneck in the design of secure and reliable integrated circuits.

In the past, people were primarily concerned with viruses [3,4] and malware [5,6], as these were widely recognized and prevalent threats. However, in recent years, attention has shifted toward hardware vulnerabilities, driven by the increasing awareness of their potential risks and high-profile attacks such as Spectre and Meltdown.

Side-channel attacks [7,8] are used to directly bypass software and target the hardware. These attacks originated in the late 1990s [9]. After the discovery of the Meltdown and Spectre attacks, most operating systems incorporated software-based countermeasures to make exploiting hardware vulnerabilities more difficult. This is because it is often very difficult and/or expensive to correct a hardware design flaw. Effective countermeasures for side-channel attacks are still under development. Security networks on a chip for mitigating side-channel attacks were studied in [10], in particular, to detect power, timing, and electromagnetic analysis attacks. In [11], a modulated power supply voltage countermeasure was developed to defeat differential and correlation power analysis side-channel attacks.

Since most side-channel attacks exploit the performance and power consumption of the hardware itself, mitigating these attacks will inevitably impact performance or even require significant changes to the system architecture, including modifying or adding new instructions. Thus, we propose a new arithmetic logic unit (ALU) architecture that integrates a random number generator and a memory address controller designed to align with the output of this ALU. The proposed design is called security ALU with memory controller (SAluMC). It integrates random numbers into operations to generate unpredictable power consumption and timing. The random numbers are also used to generate a random set number (explained in Section 3.3), which is used to store data in the cache memory.

Side-channel attacks exploit factors such as timing, temperature, and power consumption. The main objective of this work is to propose and evaluate the effectiveness of a novel architecture to counter these attacks. By introducing randomness into cache set selection, the proposed design eliminates the predictable patterns that attackers typically exploit, thereby increasing the complexity and difficulty of such attacks. In real-world scenarios, attackers often operate with limited computational and resource capabilities, so making these attacks more challenging is an effective defense mechanism. The proposed approach achieves this with minimal modifications to existing architectures, balancing enhanced security with practical implementation considerations.

The remainder of this paper is organized as follows. Section 2 discusses cache side-channel attack techniques and some approaches to thwart these attacks. Section 3 introduces the architecture and details regarding the proposed SAluMC design. Section 4 gives the experimental setup and tools used to simulate the design, and then the results are presented and discussed. Finally, some concluding remarks are given in Section 5.

## 2. Background

The increasing demand for computing power along with advances in machine learning (ML) [12,13,14] have made system-on-chip (SoC) architecture and design challenging. Consequently, attackers are finding more vulnerabilities to exploit, which has led to significant interest in side-channel attacks [15,16]. These attacks pose a significant threat to security, as unintended information leakage from hardware such as power consumption and electromagnetic emissions can be used to extract sensitive data like cryptographic keys. Given the growing concern regarding the security of private information, mitigating side-channel attacks has become an important research problem.

Modern processing unit architectures have made the power wall a problem that cannot be ignored. Therefore, designers have introduced cache into computer architectures to mitigate this problem and reduce the latency and energy consumption due to data movement. However, this creates new challenges such as race conditions, cache coherence, and cache thrashing. The cache plays a crucial role in chip data exchange, making it a prime target for side-channel attacks. As a result, attackers often focus on the cache to gather sensitive information. Thus, designers have considered cache memory countermeasures and new architectures to resist these attacks [17,18,19].

### 2.1. Cache-Based Side-Channel Attacks

Cache-based side-channel attacks exploit timing differences in CPU cache access to extract information, particularly during cryptographic operations. While the cache improves memory access speed by using temporal and spatial locality, and thus processor performance, it also creates opportunities for attackers to infer information by analyzing the timing and power consumption patterns when data are loaded or stored. Several well-known attacks have been proposed to exploit these vulnerabilities. Manipulating shared cache resources to infer memory access patterns of victim processes is a common technique that includes Flush+Reload [20] and Prime+Probe [21], which are described below. 

*Flush+Reload:*

(1) The attacker extracts the cached data at a specific location in the cache memory (Flush),
(2) waits for the target victim to execute and update the cache,
(3) reloads the memory extracted in the Flush stage, and measures and records the reload time of the cache group (Reload).

*Prime+Probe:*

(1) The attacker fills a specific cache group (Prime) with their own data,
(2) waits for the target victim to execute and update the cache,
(3) re-reads the data filled in the Prime stage, and measures and records the read time of each cache group (Probe).

In addition to these approaches, other common attack techniques are Flush+Flush [22], Evict+Time [23], Reload+Reflush [24], and Prime+Prune+Probe [25].

Existing research primarily analyzes attack techniques and their mechanisms but does not propose concrete solutions to mitigate the threats. This gap highlights the need for architectural designs that address these vulnerabilities directly. Our work aims to fill this gap by introducing a novel countermeasure that disrupts predictable patterns in cache access. This increases the complexity for attackers and mitigates the risks associated with side-channel attacks.

### 2.2. Thwarting Side-Channel Attacks

Based on the features of cache side-channel attacks, a new architecture for cache memory and a new look-up table (LUT) strategy are proposed to thwart these attacks. Random numbers have been employed in previous designs [26,27,28,29]. In [26], a random modulo cache memory design for real-time critical systems was given. An aging-robustness random modulo cache memory was proposed for probabilistically analyzable real-time systems in [27]. In [28], a random fill cache architecture was proposed to replace demand fetch with random cache fill within a configurable neighborhood window. In [29], ScatterCache was presented to thwart cache attacks through cache set randomization. However, these designs require significant changes in the cache structure or new dedicated instruction sets. Further, special supporting software is required. These greatly reduce the versatility and integrability of the design.

In addition to addressing side-channel attacks, protecting processors from hardware-level threats such as hardware Trojans (HTs) has been explored. For instance, a built-in software obfuscation methodology was proposed in [30] to minimize the exposure of sensitive information to HTs by encrypting variables, spreading them across processor registers, and introducing garbage instructions. This approach effectively obfuscates execution flow and shows the potential of runtime software manipulation to improve security.

The existing solutions discussed in this section demonstrate the variety of methods to mitigate side-channel attacks. However, they often require significant architectural changes or rely on supporting software, which can limit their scalability and practicality. In contrast, the proposed design requires minimal modifications to existing architectures, making it applicable to a wide range of systems without extensive modification. This lightweight approach ensures compatibility and ease of integration, enabling broad adoption while effectively mitigating side-channel attacks.

## 3. SAluMC Architecture

**Figure 1:**
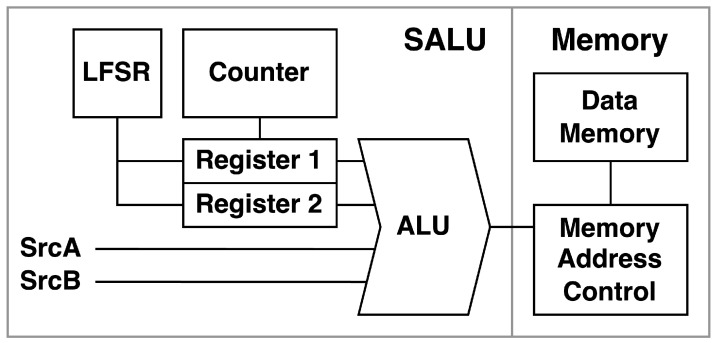
The proposed SAluMC architecture.

Figure 1 presents the architecture of the proposed design. It introduces additional functionality to the existing ALU by layering new components on top of the original architecture. This approach minimizes changes to the core architecture, ensuring compatibility and ease of integration into existing systems. To the best of our knowledge, this approach has not been considered previously.

### 3.1. SAluMC Design

To counter side-channel attacks, the new architecture integrates a redesigned arithmetic logic unit (ALU) and associated memory controller (MC), collectively known as SAluMC. It builds on the concept of using random numbers to enhance security. To increase the likelihood of widespread adoption, minimal changes are made to the core structure based on the RISC-V microarchitecture, avoiding any modification or addition of instructions. This ensures the proposed design can easily be integrated into existing systems as a direct replacement for standard ALU designs.

The proposed security arithmetic logic unit with memory controller (SAluMC) employs a linear feedback shift register (LFSR) [31,32] to generate randomness for mitigating side-channel attacks. Unlike other pseudo-random number generators (PRNGs), LFSRs offer a lightweight, efficient, and hardware-friendly solution that aligns with the ALU design. This ensures scalability while keeping the hardware requirements minimal. The advantages of the proposed design are as follows.

(1) Efficiency: The design requires minimal hardware resources and uses simple logic operations to generate pseudo-random sequences.
(2) Periodicity: The initial state and feedback connections of the LFSR can be chosen to obtain very long periods. The resulting sequences are suitably random for many applications.
(3) Flexibility: The composition and period of the random numbers can easily be changed by modifying the LFSR connections. To increase the computational complexity, a counter is employed to store the LFSR output in two registers.

The LFSR outputs stored in the registers are combined with the ALU output to form a 64-bit binary number called ALU_Result. This is passed to the memory address controller (MAC) as the data address. The MAC manages memory by storing both the data and tag based on the set numbers and loading the data according to the tag numbers.

### 3.2. The SALU Architecture

**Figure 2:**
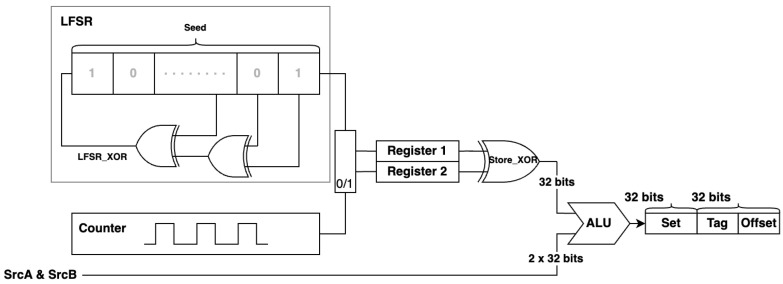
The SALU architecture.

The SALU architecture is illustrated in Figure 2. The LFSR is integrated into the SALU block to insert random numbers into the design. In the proposed design, the seed is 32 bits and is used to initialize the LFSR. The random numbers generated can be adjusted by modifying this value or modifying the LFSR connections LFSR_XOR. Note that the ALU structure largely maintains its original logic. However, simply merging the generated random numbers with the ALU results can leave the design vulnerable to attackers, who might infer operations based on calculation latency. To mitigate this risk, a binary counter is introduced based on clock cycles. When the counter is zero, the LFSR output is stored in Register 1, and when it is one, the output is stored in Register 2. Both registers are initialized to 32’b0 (32 zero bits), and the LFSR results are stored for later use.

If the control unit issues different instructions to the ALU, this will disrupt ALU operation as well as the interaction between the two registers. For example, when executing a store instruction (SW), which adds the immediate value (offset) to the contents of the base register within the ALU block, we introduce timing and power consumption variations by performing an XOR operation between the contents of the two registers. The result, denoted Store_XOR, is a 32-bit number that helps disrupt predictable patterns and increases resistance to side-channel attacks. Store_XOR serves as the set in the cache memory, while the ALU result functions as the tag and offset. The left half of the 64-bit output (bits 33 to 64) is Store_XOR, while the right half (bits 1 to 32) contains the ALU result. Together, these 64 bits form the complete output of the SALU block, and this is passed to the MAC for further processing.

### 3.3. Memory and Address Controller

**Figure 3:**
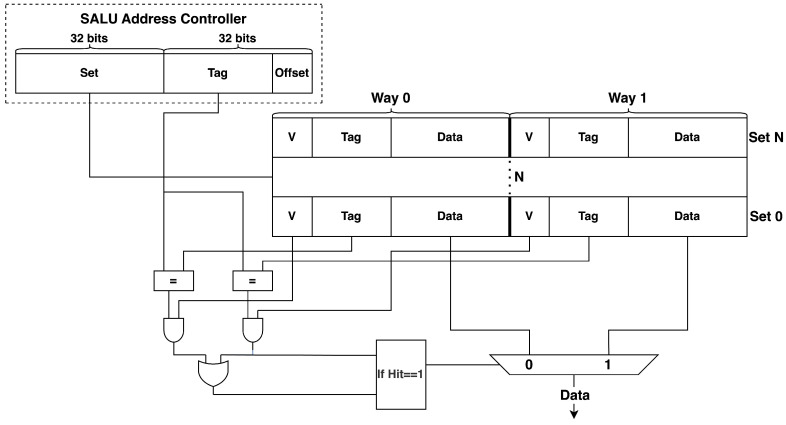
The memory controller architecture.

For load or store instructions, the SALU result is forwarded to the MAC as shown in Figure 3. This figure presents the memory structure in two ways, but any structure can be employed based on the required dimensions. Initially, all the valid bits (as shown by V in Figure 3) are set to 0. If a store instruction (SW) is received from the instruction memory, a set number is generated using the random numbers from the SALU address controller to perform the modulo operation to store data. The 32-bit width of the set field generates a value that is often much larger than the actual number of cache sets available. To account for this and balance universality with hardware efficiency, we do not modify the length of the set number in the SALU address controller. Instead, the larger set number is taken modulo the number of cache sets to obtain a valid set number. However, modulo operations can be computationally complex and costly in terms of hardware and energy, so bit-shifting is used to determine the target set number. Once this number is determined, the controller checks whether the corresponding blocks are available. If the block under the target set is empty, the tag and data are stored in that set, and the valid bit is set to 1. If Way 0 is full, the controller checks Way 1, and if it is available, stores the tag and data there and sets the valid bit to 1. If both ways are full, the data in Way 0 are replaced.

64-bit data are also received from the SALU block for load instructions. Figure 3 shows that we need to determine whether the tag contained in the address can be found in the memory. If the target tag is already in Way 0 and the valid number is 1, the hit signal is set to 1, and the data are loaded from Way 0 to the on-core register, and vice versa. If the target tag is not already in memory in either Way 0 or Way 1, or if the valid bit is 0, the memory outputs a miss signal to request loading the required data from main memory.

Since load operations (LW) do not require a set number, the random set number does not impact the loading process. However, during store operations (SW), the presence of random numbers makes it difficult for attackers to predict which specific cache set is being used. This adds a layer of defense against attacks that depend on targeting specific cache sets, effectively thwarting such attempts.

## 4. Design Evaluation

This section presents a comprehensive analysis of the design through simulation-based experiments. The experimental setup, design architecture, performance evaluation, and set number probabilities are given. Examining both the theoretical foundation and practical implications of the design confirms how the integration of randomness in cache set selection increases attack complexity while maintaining compatibility with existing systems. Results are also given to validate the feasibility and robustness of the architecture under realistic conditions.

### 4.1. Experimental Setup

The design and verification process has two stages. First, the pseudo-random number generator was designed using a Python 3.10.12 simulator. Then, the RTL design was implemented using the SystemVerilog hardware description language. The Xilinx Vivado 2018.3 software suite and a Xilinx ZYNQ 7020 field-programmable gate array (FPGA) board were employed for validation and analysis. This involved integrating the SAluMC design into the classic five-stage pipeline architecture based on the RISC-V instruction set. The design was synthesized using the 45 nm FreePDK45 EDA library and the Synopsys Design Compiler R-2020.09-SP4 was used for performance evaluation.

### 4.2. Design Architecture

**Figure 4:**
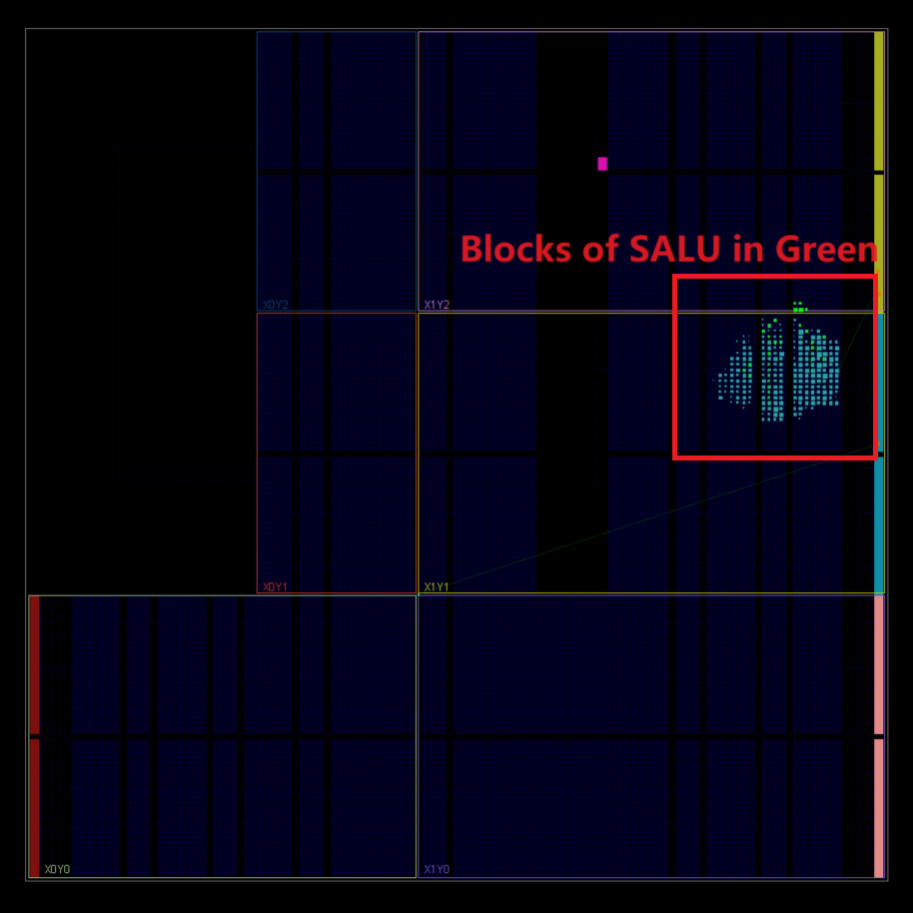
The SAluMC device architecture in the RISC-V microarchitecture of the five-stage pipeline.

The ZYNQ 7000 family is built on the Xilinx SoC architecture and was introduced in April 2010. This FPGA family integrates two ARM Cortex-A9 cores in a processing system. The programmable logic section of this FPGA board is based on Xilinx 7-series FPGAs utilizing 28 nm process technology [33]. Figure 4 and Table 1 show that the proposed design uses only 1.57% (837 out of 53,200) of the overall LUT resources. In this figure, green indicates the SALU architecture implementation across the entire RISC-V-based five-stage pipeline microarchitecture, which is about 4.1% of the total LUT usage.

**Figure 5:**
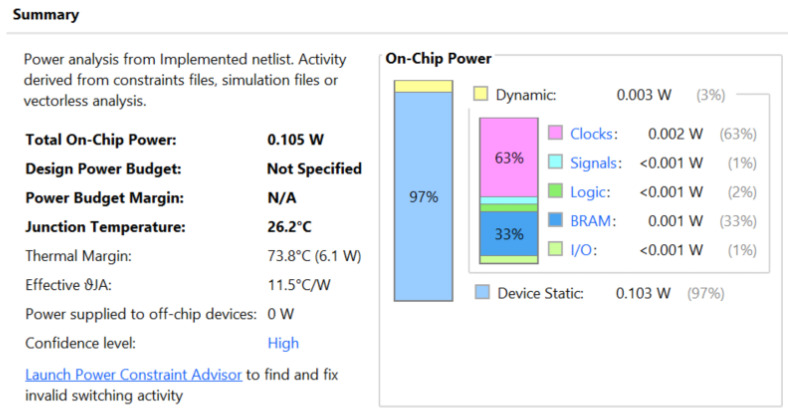
On-chip power for SAluMC with the five-stage pipeline core.

Figure 5 gives the on-chip power consumption of the proposed design executed on the 7200 series FPGA board at temperature. This indicates that the power consumption for the static area is only 0.103 W even though it constitutes 97% of the overall power consumption. This may be because only 128 instructions were used to test the design on the FPGA board. The high confidence level reported reflects the highest behavior of the Vivado software during power consumption simulation.

### 4.3. Performance Analysis

In this section, the power consumption, area cost, and layout of the SALU architecture are evaluated using the Vivado software, and SALU performance is compared with that of the original ALU design.

**Figure 6:**
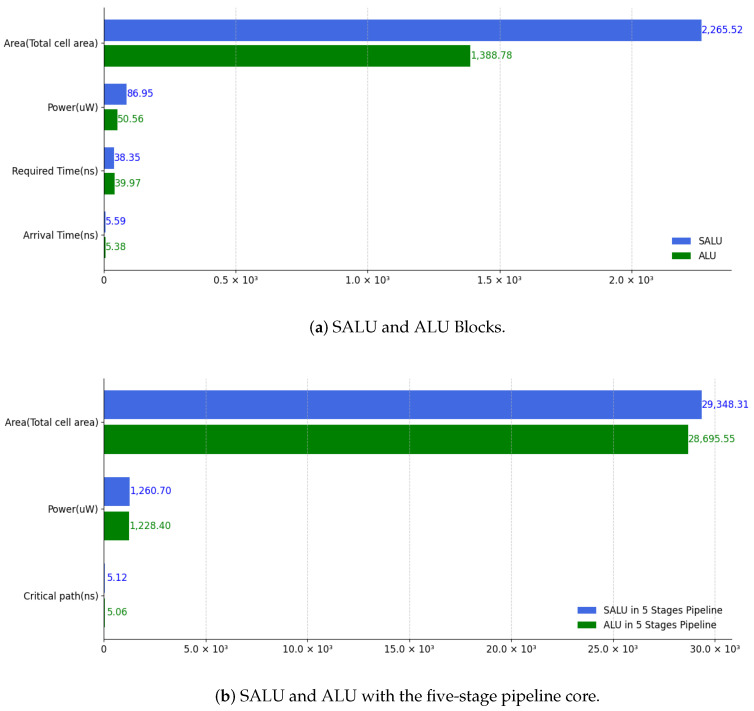
SALU and ALU performance.

*(1) SALU design performance:* Figure 6 presents the area, power, and timing consumption of the SALU and original ALU blocks, and also the SALU and ALU after integration into the RISC-V five-stage pipeline microarchitecture. These results were obtained using the Synopsys Design Compiler with the same library and constraints for consistency. Since the SALU integrates more registers and blocks compared to the original ALU, it results in higher area, power, and timing costs. However, as a key design goal is minimal modification of the original architecture, only a small number of registers were employed to simplify the design and reduce complexity while achieving the desired functionality. Figure 6a shows that the size of the SALU is 1.63 times larger than the original ALU, with power consumption 1.71 times higher and timing cost increased by a factor of 1.04. Considering that the ALU is just one component of the system within the RISC-V microarchitecture, the additional resources, when distributed across the entire system, as shown in Figure 6b, are acceptable. Furthermore, in some cases, including registers in the SALU design can assist with clock synchronization, which can help reduce static power consumption.

**Figure 7:**
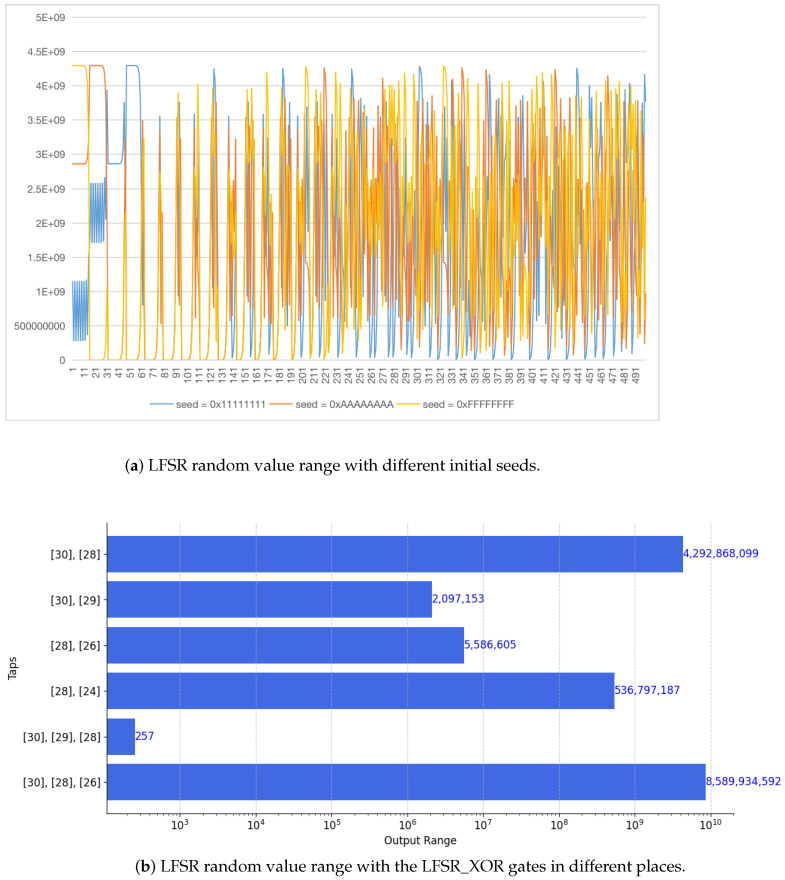
LFSR random value range with different initial seeds and structures.

*(2) LFSR structure:* As mentioned above, one of the reasons an LFSR was chosen for the SAluMC design is that it is reconfigurable. However, employing pseudo-random numbers can degrade the performance of on-chip memory. Therefore, the range of the pseudo-random numbers should be chosen to satisfy the requirements. This is achieved via the seed and the number and position of the XOR gates in the LFSR block. Figure 7a gives the distribution of the pseudo-random numbers generated using different seed values. This shows that a change in the seed results in a significant change in the numbers generated. This makes it very difficult for an attacker to predict the numbers. Figure 7b gives the range of the pseudo-random numbers with different values of LFSR_XOR. A log scale is used to better illustrate the changes in range. This figure shows that only changing the location of the XOR gates (even with only one gate) has a significant effect on the number range.

**Figure 8:**
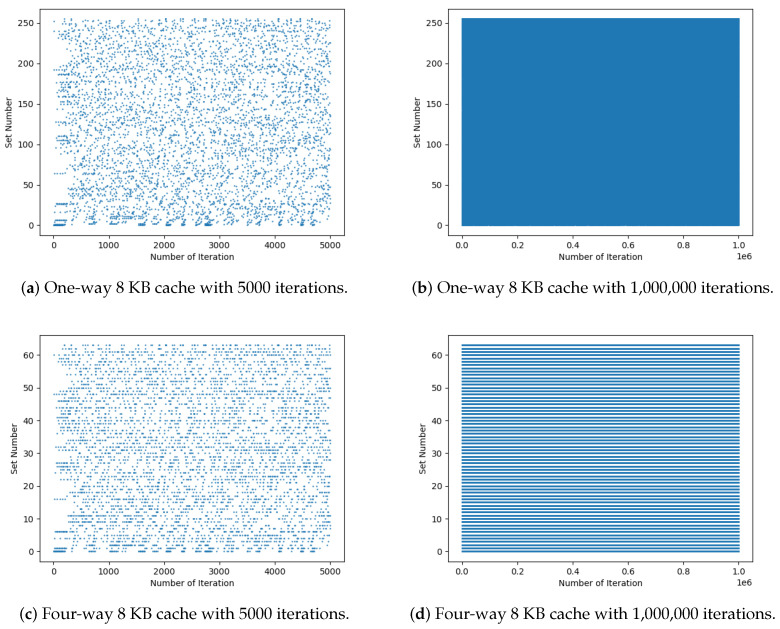
Set numbers for one-way and four-way 8 KB cache sizes with 5000 and 1,000,000 iterations.

**Figure 9:**
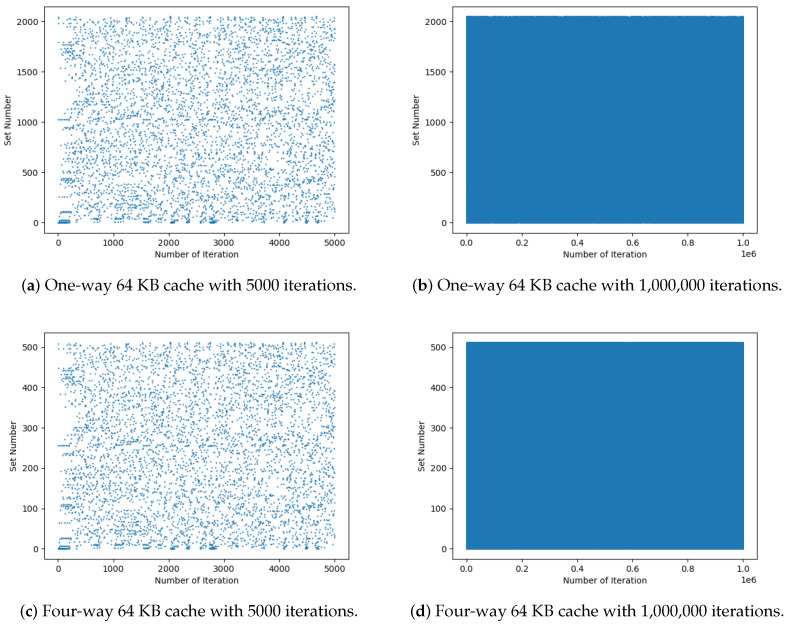
Set numbers for one-way and four-way 64 KB cache sizes with 5000 and 1,000,000 iterations.

*(3) Distribution of set numbers for different numbers of iterations:* Random numbers are the main method of protecting the architecture presented in Section 3 against side-channel attacks. This approach creates variations in power consumption during calculations in the SALU as the random numbers are used to store the results in cache memory. Therefore, the coverage of the random numbers for all sets should be examined. Figure 8 and Figure 9 illustrate the set numbers for 5000 and $10^{6}$ iterations. To better compare the distributions, cache sizes of 8 KB and 64 KB were used as they are commonly used in modern CPUs. Further, one and four ways were considered. The one-way and four-way associativities illustrated in Figure 8 and Figure 9 were obtained using the memory address controller shown in Figure 3. In standard cache designs, a set number may map to multiple cache ways, such as in a four-way associative cache. After identifying the set number, the tag and validation bit (V) are used to locate the specific way. A valid bit (V = 1) ensures the data are valid, while V = 0 indicates invalid data that cannot be used. This provides flexibility in handling multiple ways for the same set number. Figure 8a and Figure 9a give the results for 5000 iterations and indicate that the range of set numbers for 64 KB is larger than for 8 KB. Thus, Figure 9a appears more sparse, but the numbers still provide random coverage of the range. Figure 8b and Figure 9b show that, for $10^{6}$ iterations, there is excellent coverage of the set numbers. Although some unevenness can be seen in Figure 9b, the coverage is sufficiently random. This randomness will make it difficult for attackers to predict the set number where data are stored. When the number of ways is increased from 1 to 4, which is more typical, Figure 8c and Figure 9c show the set number distributions for 5000 iterations and Figure 8d and Figure 9d show the distributions for $10^{6}$ iterations. Figure 8d indicates that an increase in the number of ways increases the number of sets so it appears denser, but the randomness is similar.

**Figure 10:**
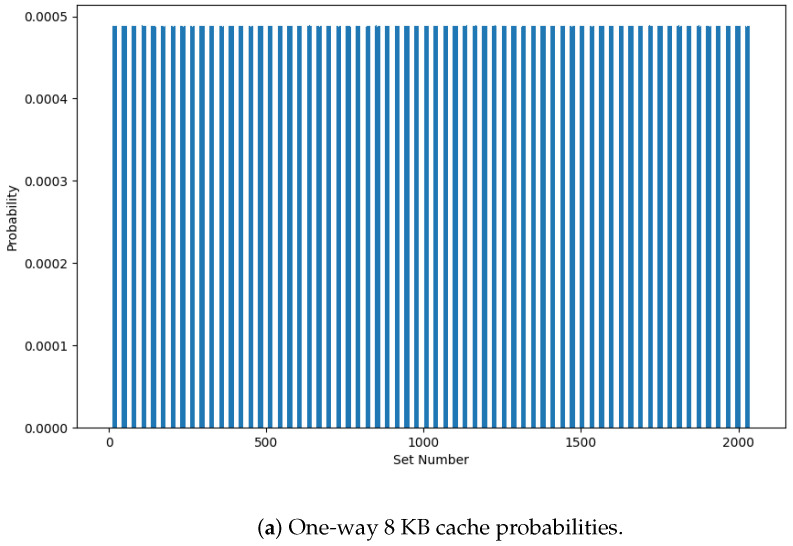

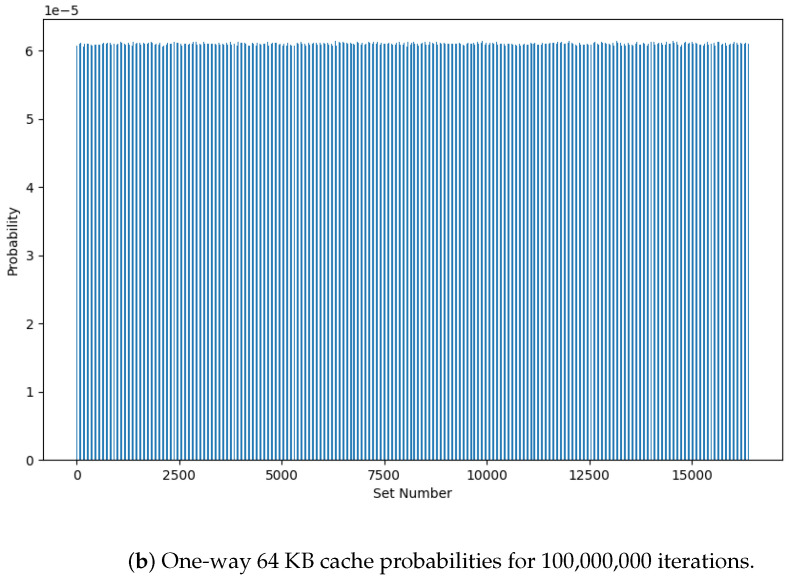
Set number probabilities for one-way 8 KB and 64 KB caches.

*(4) Set number probabilities:* Figure 10 gives the probability distributions of the set numbers for one-way 8 KB and 64 KB caches with 100,000,000 iterations. This confirms that the set number distribution is nearly uniform, indicating a balanced use of all cache sets. While the distribution for the 64 KB cache has slight variations due to the large number of sets, this will not compromise the effectiveness of the randomization. The randomness will significantly complicate potential side-channel attacks that rely on predictable patterns in cache behavior, as all set numbers have approximately the same probability of occurrence. Thus, the LFSR output is effective in creating random storage locations for data within cache sets. This significantly reduces the likelihood of an attacker obtaining data from a particular cache set.

### 4.4. Impact of Cache Set Distribution on Attack Complexity

The results given above demonstrate the security benefits of randomizing cache set selection. The number of sets will impact the complexity of attacks depending on the application and system configuration. However, increasing the number of sets will increase this complexity and make timing correlations less predictable.

In real-world scenarios, attackers often have limited resources and thus only a small number of measurements to deduce patterns. Distributing data across multiple sets forces attackers to obtain a broader range of measurements, significantly increasing the difficulty of identifying consistent patterns. While this makes timing attacks less feasible, different configurations may be required for specific applications, so security must be balanced with system performance.

### 4.5. Empirical Validation

While the proposed architecture has strong theoretical potential to mitigate side-channel attacks, empirical validation through real-world attack implementation should be conducted. The evaluation presented is based on theoretical security principles, focusing on how the introduction of randomness disrupts predictable patterns in cache access and power consumption, which are typically exploited in timing and power analysis-based attacks. Implementing and testing these attacks requires specialized hardware, controlled environments, and extensive resources. Due to these constraints, it was not possible to conduct such experiments within the scope of this work. While the mitigation of side-channel attacks is well-supported by the results presented, practical validation remains an important next step. Future work will focus on implementing real-world scenarios to empirically evaluate the effectiveness of the proposed approach. This will provide comprehensive insights into the architecture resilience against practical side-channel attacks and help identify potential areas for further optimization.

## 5. Conclusions

Electronic devices are now essential in daily life, and they contain chips that are critical to their operation. This dependency creates opportunities for attackers to exploit vulnerabilities in both software and hardware to access user information. Hardware vulnerabilities, particularly those targeted by side-channel attacks, are especially difficult to detect and mitigate. Despite significant research efforts to develop solutions, eliminating these attacks remains challenging. As computing power increases along with SoC integration, cache memory has become increasingly important. It is a fundamental component of most computing systems and thus has been the focus of extensive research aimed at optimizing its structure, data transmission, and storage strategies. However, side-channel attacks on the cache remain a serious concern.

This paper introduced a novel ALU structure and an accompanying MC aimed at mitigating side-channel attacks such as Prime+Probe and Flush+Reload that exploit specific cache sets. The proposed approach generates random numbers within the ALU block to store data in random cache sets, thereby preventing attackers from inferring user data through predictable cache behavior. However, this randomization can affect memory temporal and spatial locality, potentially impacting cache performance. While techniques such as increasing cache associativity or storing similarly attributed data can help mitigate these drawbacks, they do not completely resolve the issues. Future work will focus on improving memory and loading strategies using randomization. Methods will be developed to preserve locality and thus minimize performance degradation while enhancing the effectiveness of side-channel attack mitigation techniques.

## Figures and Tables

**Table 1:** Hierarchy for the five-stage pipeline integrated with SAluMC.

Instance	Module	Total LUTs	Logic LUTs	LUTRAMs	SRLs	FFs	RAMB36	RAMB18	DSP48 Blocks
RISC_pip	(top)	837	755	80	2	529	0	0	0
(RISC_pip)	(top)	0	0	0	0	0	0	0	0
DataMEM	dmem	32	0	32	0	0	0	0	0
RISC_PIP	riscv_pip	805	755	48	2	529	0	0	0
ControlU	controller	7	7	0	0	20	0	0	0
c_pipregDM_WB	c_IM_IW	0	0	0	0	3	0	0	0
c_pipregEX_DM	c_IEx_IM	0	0	0	0	5	0	0	0
c_pipregID_EX	c_ID_IEx	7	7	0	0	12	0	0	0
DataPath	datapath	798	748	48	2	509	0	0	0
IF	flopenr	69	69	0	0	32	0	0	0
PCaddBranch	adder	0	0	0	0	0	0	0	0
PipRegDM_WB	IMem_IW	107	107	0	0	101	0	0	0
PipRegEX_DM	IEx_IMem	1	1	0	0	101	0	0	0
PipRegID_EX	ID_IEx	425	425	0	0	164	0	0	0
PipRegIF_ID	IF_ID	100	100	0	0	87	0	0	0
RegFile	regfile	64	16	48	0	0	0	0	0
SALU	SALU	33	31	0	2	24	0	0	0
(SALU)	SALU	25	25	0	0	2	0	0	0
counter	counter	1	1	0	0	1	0	0	0
lfsr	lfsr	7	5	0	2	21	0	0	0

**Note:** The number of lower-level cells may be larger than the number of parent cells due to cross-hierarchy LUT combining.

## Data Availability

No new data were created or analyzed in this study. Data sharing is not applicable to this article.

## Abbreviations

The following abbreviations are used in this manuscript:

ALU	Arithmetic logic unit
MAC	Memory address controller
MC	Memory controller
ECU	Engine control unit
SAluMC	Security ALU with memory controller
ML	Machine learning
SoC	System-on-chip
SW	Store instruction
LW	Load operation
LUT	Look-up table
LFSR	Linear feedback shift register
XOR	Exclusive OR
FPGA	Field programmable gate array
